# Oral vaccination with recombinant *Lactobacillus casei* expressing Aha1 fused with CTB as an adjuvant against *Aeromonas veronii* in common carp (*Cyprinus carpio*)

**DOI:** 10.1186/s12934-022-01839-9

**Published:** 2022-06-13

**Authors:** Chong Chen, Shuo Zu, Dongxing Zhang, Zelin Zhao, Yalu Ji, Hengyu Xi, Xiaofeng Shan, Aidong Qian, Wenyu Han, Jingmin Gu

**Affiliations:** 1 State Key Laboratory for Zoonotic Diseases, Key Laboratory of Zoonosis Research, Ministry of Education, College of Veterinary Medicine, Jilin University, Changchun, 130062 People’s Republic of China; 2 Key Laboratory of Bionic Engineering, Ministry of Education, Jilin University, Changchun, 130025 People’s Republic of China; 3 College of Animal Science and Technology, Jilin Agricultural University, Changchun, 130118 People’s Republic of China; 4 Jiangsu Co-Innovation Center for the Prevention and Control of Important Animal Infectious Diseases and Zoonoses, Yangzhou University, Yangzhou, 225009 People’s Republic of China

**Keywords:** *Aeromonas veronii*, Lactic acid bacteria, CTB, Mucosal immune adjuvant

## Abstract

*Aeromonas veronii (A. veronii)* is a pathogenic that can infect human, animal and aquatic organisms, in which poses a huge threat to the health of many aquatic organisms such as *Cyprinus carpio*. In this study, *Lactobacillus casei (L. casei)* strain CC16 was used as antigen deliver carrier and fused with cholera toxin B subunit (CTB) as an adjuvant to construct the recombinant *L. casei* pPG-Aha1/Lc CC16(surface-displayed) and pPG-Aha1-CTB/Lc CC16(surface-displayed) expressing Aha1 protein of *A. veronii*, respectively. And the immune responses in *Cyprinus carpio* by oral route was explored. Our results demonstrated that the recombinant strains could stimulate high serum specific antibody immunoglobulin M (IgM) and induce a stronger acid phosphatase (ACP), alkaline phosphatase (AKP), C3, C4, lysozyme (LZM), Lectin and superoxide dismutase (SOD) activity in *Cyprinus carpio* compared with control groups. Meanwhile, the expression of Interleukin-10 (IL-10), Interleukin-1β (IL-1β), Tumor Necrosis Factor-α (TNF-α), immunoglobulin Z1 (IgZ1) and immunoglobulin Z2 (IgZ2) in the tissues were significantly upregulated compared with Lc-pPG or PBS groups, indicating that humoral and cell immune response were triggered. Additionally, recombinant *L. casei* could survive and colonize in fish intestine. Significantly, recombinant *L. casei* provides immune protection against *A. veronii* infection, which *Cyprinus carpio* received pPG-Aha1-CTB/Lc CC16 (64.29%) and pPG-Aha1/Lc CC16 (53.57%) had higher survival rates compared with the controls. Thus, we demonstrated that recombinant pPG-Aha1/Lc CC16 and pPG-Aha1-CTB/Lc CC16 may be the promising strategy for the development of an oral vaccine against *A. veronii*.

**Supplementary Information:**

The online version contains supplementary material available at 10.1186/s12934-022-01839-9.

## Introduction

*Aeromonas veronii (A. veronii),* an opportunistic pathogen, is a member of the genus Aeromonas, which is among the utmost frequent bacteria in aquatic environment and widely distributed in freshwater, estuarine environments, drinking waters, wastewaters, and sewage [1, 2]. It is mainly associated with diseases in aquatic animals including common carp (*Cyprinus carpio*) [3], tilapia (*Oreochromis mossambicus*) [4] and northern snakehead (*Channa argus*) [5], leading to serious economic losses in aquaculture. Simultaneously, *A. veronii* can infect human and mammals, which caused meningitis, sepsis, diarrhea and wound infection, and resulted in serious threat to human public health security [6–8].

Currently, the treatment of some bacterial diseases in aquaculture is mainly based on antibiotics. Although antibiotics have the advantages of low cost, high efficiency, and convenience, it still has several shortcomings, such as bacterial resistance and residues of antibiotics in cultured aquatic products [9]. Therefore, vaccines against *A. veronii* have been reported successively included inactivated vaccines, DNA vaccines, attenuated vaccines and oral vaccines [10–13]. However, outer membrane proteins (OMPs), including outer membrane protein A (OmpA), porins, lipoprotein (Lipoprotein, Lpp), play potentially crucial roles in antibiotic resistance, particularly in Gram-negative bacteria [14]. OMPs are the interface between the host and the pathogen, which can be easily recognized by the immune system of the host [15]. And porin (AMS64_15290) belongs to the family of gram-negative bacteria porins, such as aha of OMPs [16]. Previous studies have shown that Aha1 of *Aeromonas hydrophila* had high immunogenicity which played an important role in adhesin and virulence [17, 18]. Therefore, it was a potential candidate protective antigen.

Lactic acid bacteria (LAB), a type of Gram-positive and non-sporulating bacteria which has low Guanine and Cytosine (GC) content, are highly related to human and animal health. They are ‘generally regarded as safe (GRAS)’ microorganisms, used in industries closely related to human life such as agricultural production and animal husbandry [19]. LAB can colonize the surface of the intestinal mucosa and are recognized as a "food-grade" antigen deliver carrier [20]. Over the past two decades, LAB have several attractive advantages as a mucosal vaccine carrier, such as an excellent safety profile for human use, absence of endotoxins, resistance to low pH and intrinsic adjuvanticity [21]. Meanwhile, probiotics can overcome some limitations of attenuated vaccines as mucosal delivery carrier and fill the requirements of delivery systems for mucosal administration [22]. Compare with other mucosal delivery systems, LAB have become potential candidates.

Recently, mucosal immunization has attracted much interest as a means of generating protective immunity against mucosal pathogens. In contrast, only few mucosal vaccines are approved for human [23]. The development of a broad range of mucosal vaccines will necessitate the development of safe and effective mucosal adjuvants and delivery systems. Presently, common mucosal adjuvants include ADP-ribosylated enterotoxin (CT), synthetic oligodeoxynucleotides (CpG ODN) containing unmethylated CpG dinucleotides and monophosphoryl lipid A [24]. The attractive and powerful mucosal adjuvants are non-toxic derivatives of cholera toxin (CT), and much effort and significant progress have been made recently to generate toxicologically acceptable derivatives of these toxins with retained adjuvant activity, in which among these are the non-toxic, cholera toxin B-subunit (CTB) [25, 26]. It can enhance the immunogenicity of antigens, and induce local mucosal immune responses through stimulating and activating mucosal-related cells, and they have great potential as adjuvants for mucosal immune antigens [27]. Since mucosal adjuvants play an attractive role in enhancing immune response, it is necessary to choose a suitable mucosal immune adjuvant.

In this study, we have accordingly taken advantage of the safety of CTB as an adjuvant and fused with target antigen Aha1 of *A. veronii* to devise a vaccine by constructing the genetically engineered recombinant *L. casei* pPG-Aha1/Lc CC16 and pPG-Aha1-CTB/Lc CC16, respectively. To evaluate the effects of the immunized *Cyprinus carpio* via oral administration route, we detected the colonization in the fish intestine and protective efficacy of the recombinant *L. casei* against *A. veronii* infection. The results would provide a theoretical basis for the development of oral *L. casei* delivery vector vaccines and the application of mucosal immune adjuvants in the aquaculture industry.

## Materials and methods

### Experimental fish and ethics statement

Health *Cyprinus carpio* individuals (n = 280, average weight 60 ± 1 g and length 13.76 ± 0.25 cm) without specific pathogen-free were supplied by a fish farm (Changchun, Jilin, China). Details of all *Cyprinus carpio* were listed in Additional file 1: Table S1. Fish were kept at 24 ± 2 ℃ in closed recirculating water tanks (120 cm × 50 cm × 60 cm), with natural photoperiod. To ensure the reliability of this experiment, we fed with a diet of commercial dry pellets twice a day which the feeding rate of 1.5% body weight. During the whole experiment periods, the tanks were cleaned once five days and one-third of the water was replaced three days. All experimental procedures for this study were in accordance with the Regulations for Animal Experimentation of Jilin Agriculture University (JLAU08201409).

### Vectors, bacterial strains and growth conditions

The *Escherichia coli*-*Lactobacillus* shuttle vector pPG which contain an anchoring matrix-encoding pgsA gene derived from *Bacillus subtilis* behind the target gene was kindly provided by Dr. Zhang (Jilin Agricultural University, China) [28]. *L. casei* CC16 (isolated from the intestine of *Cyprinus carpio*) was a plasmid-free strain, which was cultivated statically in De Man, Rogosa and Sharpe (MRS) medium at 30 ℃, and *Escherichia coli* competent cells MC1061 was cultivated in Luria–Bertani (LB) medium at 37 ℃ with shaking. *L. casei* CC16 and *E. coli* competent cells MC1061 were donated by the key laboratory of Animal Production College of Animal Science and Technology, Jilin Agricultural and the concentration of Chloramphenicol (Cm; Sigma, USA) was utilized at 10 μg/mL. *A. veronii* TH0426 (isolated from *Pelteobagrus fulvidraco*) was a kind gift from Dr. Kang of Jilin Agricultural University [29], which was cultivated in Rimler-Shotts (RS) medium at 30 ℃.

### Construction of recombinant *E. coli* and *L. casei*

The Aha1 (1038 bp) gene of the *A. veronii* TH0426 strain (Genbank: CP012504.1) was amplified by the primer sets Aha1-F/Aha1-R with *Sma*I or *Eco*RV sites which shown in Table 1 and gel purified. And the Aha1 gene was fused with the CTB fragment by combining with a flexible connection linker (GGGGS)3 was amplified by the primer sets Aha1-F1/Aha1-R1 and CTB-F/CTB-R with *Spe*I or *Eco*RV sites (Table 1) and gel purified. The polymerase chain reaction (PCR) product of Aha1 gene or Aha1-CTB fused gene was cut with *Sma*I/*Eco*RV and *Spe*I/*Eco*RV restriction sites and inserted into the corresponding sites of pPG to generate pPG-Aha1 and pPG-Aha1-CTB, respectively. Finally, these recombinant plasmids were introduced into *L. casei* by electroporation as described previously [30]. The integrity of generation of recombinant *L. casei* at each step of the process was verified by sequence analysis. And pPG plasmid, as a negative control was introduced into *L. casei* by electroporation for the subsequent works.

**Table 1:** Details of primer sequences used in this study

Primer	Sequence (5′ → 3′))	Annealing temperature	Accession number
IL-10	F: GATTGTGGCCATGATGACTTG R: GTAGTGTGATGGAATGGTGATGTG	61 °C	AB110780
IL-1β	F: GCCGACTCTGATGAACTGGA R: TGCTGTGAGGATGCGAAGA	61 °C	AB010701
TNF-α	F: AGGTGATGGTGTCGAGGAGGAAG R: AGACTTGTTGAGCGTGAAGCAGAC	60 °C	AJ311800
IgZ1	F: AGTGCTCCCATCCATCAAAAC R: GTGGTGCTGAGCTTTTCATACTCTT	62 °C	AB598367.1
IgZ2	F: ACAAGTGTCAACAAGCAAAAGTGAG R: AGAGGAAGAGGAAGATGAATGAGGT	62 °C	AB598368.1
β-actin	F: CGTGATGGACTCTGGTGATG R: TCGGCTGTGGTGGTGAAG	60 °C	M24113
Aha1	F: CCCCCGGGATGAAAAAGACAATTCTGGCTAT R: AAGATATCTTAGAAGTTGTACTGCAGAGCAACA	53 °C	CP012504.1
CTB	F: GTACAACTTCGGAGGTTCAGGAGGCAGC R: CGGATATCTTAATTTGCCATACTAATTGCG	55 °C	HM590452.1
pPG-1	F: GCTGCTTTACTTGCTGTTG R: GATTGCCCAAGTCCGTTCC	62 °C	
dnaA	F: TCTGTTTATTTATGGTGGCG R: CTGCGGTCATCAAGTTTCA	54 °C	

### Western blot assay

Western blotting was used to detect the expression of recombinant Aha1 gene or recombinant Aha1-CTB as described previously [31]. Recombinant *L. casei* pPG-Aha1/Lc CC16, pPG-Aha1-CTB/Lc CC16 and pPG/Lc CC16 were cultured statically in MRS medium containing 10 μg/mL of chloramphenicol at 30 ℃ for overnight. Then, xylose was supplemented with the culture medium containing 10 g/L (final concentration) to induce the expression of antigen. Subsequently, the gel from 12% sodium dodecyl sulphate–polyacrylamide gel electrophoresis (SDS-PAGE) was transferred to nitrocellulose membrane and mouse anti-Aha1 antibody (previously prepared in our laboratory) was used as primary antibody at 1:100 ratio and horseradish peroxidase (HRP)-conjugated goat anti-mouse IgG (Sigma, USA) diluted at 1:200 ratio. Finally, the target protein was visualized by chemiluminescence and was detected using Western ECL substrate (Termo Scientific) in an Amersham Imager 600 (GEHealthcare, UK).

### Immunofluorescence analysis

For evaluating the surface expression, recombinant strain pPG-Aha1/Lc CC16, pPG-Aha1-CTB/Lc CC16 and control strain pPG/Lc CC16 was harvested as previously modified and described condition [32], and resuspended in 1% bovine serum albumin (BSA) containing mouse anti-Aha1 antibody (1:100) and incubated for 1 h at 37 ℃. Subsequently, bacteria and antibody complex were washed with phosphate buffered saline with tween 20 (PBST) for five times and treated with fluorescein isothiocyanate (FITC)-conjugated anti-mouse immunoglobulin G (IgG) antibodies (1:1000) (Sigma, USA) containing 1% Evans blue for 2 h avoiding light contact. Cells were washed five times using PBST, streaking on a glass slide, air-dried and heat-fixed. Immunofluorescence analysis was performed by the fluorescence microscopy (Zeiss LSM710).

### Stability of recombinant *L. casei* strains

The stability of recombinant *L. casei* strains was detected as previously described by Song, 2014 [33]. In brief, the culture of recombinant *L. casei* strains (1% inoculum) were continuously transferred 50 generations at interval of 12 h in MRS medium supplemented with 10 μg/mL of chloramphenicol at 30 ℃, then genomic DNA of each generation was extracted and detected by PCR for the presence of target fragment using specific primers for the target gene.

### Fish immunization and sample collection

**Fig. 1:**
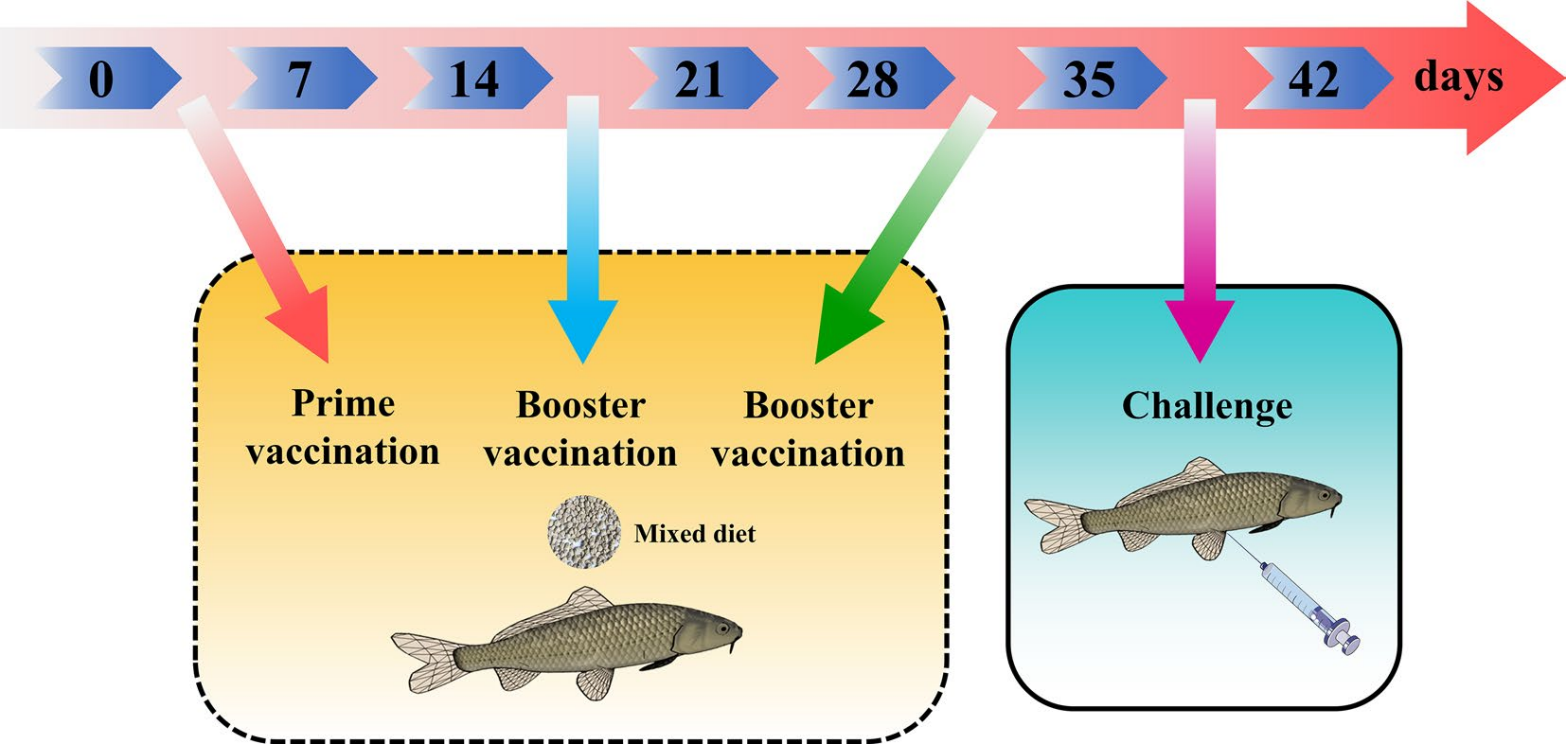
Experimental schedule. The oral immunization was conducted three times with an interval of 14 days. Prime vaccination was given between days 1–3, booster vaccination was given between days 15–17, second booster vaccination was given between days 29–31 and challenge was carried out on day 36

The recombinant *L. casei* strains were cultivated in MRS supplemented with 10 μg/mL of chloramphenicol and contained 2% xylose. Then, 200 mL of overnight cultures were thoroughly mixed with 20 g of commercial diet feed which were oven-dried at 38 ℃ for 8 h. Finally, the diet feed contained an average of 10^9^ colony forming unit (CFU)/g and kept at 4 ℃ prior to feeding. The fish individuals were divided into four groups (n = 70 per group) randomly, and orally fed with the mixed feed containing pPG-Aha1/Lc CC16, pPG-Aha1-CTB/Lc CC16, pPG/Lc CC16 (negative control) and phosphate buffered saline (PBS, Blank control) on days 1, 15 and 29, respectively and the immune protocol was administered on three consecutive days at days 1–3 (prime vaccination), 15–17 (booster vaccination), 29–31 (second booster vaccination) and 36 (challenger) (Fig. 1). All samples were processed as previously described [34, 35]. Three *Cyprinus carpio* individuals were randomly selected from each group on days 0, 7, 14, 21, 28, 35 and 42. Afterwards, fish were anaesthetized by bath immersion with 60 mg/L MS-222 (Sigma, USA) for 5 min before sampling. Blood, liver, spleen, head kidney (HK), intestine and gills were aseptically collected, and stored at – 80 ℃ for the subsequent works. Total RNA was isolated from the above samples using Trizol reagent (B610409) according to the manufacturer's protocols (Sangon Biotech, China), then cDNA synthesis was performed as described previously [36].

### Antigen-specific IgM and non-specific immune enzymes

The levels of Aha1-specific antibody immunoglobulin M (IgM) in the serum was detected by Enzyme-linked immunosorbent assay (ELISA), as described previously [37]. In brief, 96-well microtiter plates were coated 12 h at 4 ℃ with the recombinant 5 μg/mL Aha1 protein and followed blocking with 2% BSA for 2 h at 37 °C. Then, five microliters per well of serum sample were added and incubated for 1 h at 37 ℃. Bound antibodies were tested using HRP-conjugated anti-common carp IgM monoclonal antibody (Stirling, Scotland) incubated at 37 ℃ for 1 h and the absorbance was measured at 450 nm. To evaluate the activity of non-specific immune enzymes, acid phosphatase (ACP, J69003-A), alkaline phosphatase (AKP, J6346-A), C3 (J69024-A), C4 (J6301-A), lysozyme (LZM, J69259-A), Lectin (J69099-A) or superoxide dismutase (SOD, J3822-A) were examined by using ELISA kits (Jingmei Biotechnology, China) following the manufacturer' s instructions. All analyses were performed in triplicate.

### Determination of immune related genes expression

The mRNA levels of immune related genes (IL-10, IL-1β, TNF-α, IgZ1 and IgZ2) were analyzed in different tissues using Real-Time PCR as described in the previous publication by Cao., 2020 [38]. The qRT-PCR primers for selected immune genes were designed based on the conserved regions of the common carp Gene Bank sequences, were presented in Table 1. β-actin gene, as the housekeeping gene, to normalize the expression of the target genes. The total volume of RT reaction of 20 μl including 10 μl of 2 × SYBR Green Master Mix (Takara, Japan), 8 μl of sterile water, 1 μl of 1000 ng of total input RNA sample, and 0.5 μl of both forward and reverse specific primers. The stratagene MxPro software (strata-gene mx3005p, USA) was used for data analysis. All qRT-PCR were conducted with three replicates.

### Colonization ability of recombinant *L. casei* in the fish intestine

Briefly, groups of three fish each were sacrificed on days 1, 3, 7, 12 and 18, then the foregut, midgut and hindgut were extracted and cut longitudinally under aseptic conditions. Subsequently, the collected intestines were homogenized aseptically in PBS containing 1% fetal calf serum according to published protocols, and 100 μl of 10^4^-fold diluted samples were plated on MRS agar plates supplemented with 10 μg/mL chloramphenicol and incubated anaerobically for 24 h at 30 ℃ [39]. Next, the single colonies were randomly selected and conducted to PCR using pPG-vector specific primers and *L. casei* housekeeping gene *dnaA* primers in Table 1 as previously described by Cheung et al. [40]. Initial denaturation was at 94 ℃ for 5 min, followed by 30 cycles of denaturation at 94 ℃ for 1 min, annealing at 55 ℃ for 1 min, and extension at 72 ℃ for 10 min. PCR products were confirmed by sequence analysis.

### Challenge test

To evaluate the protective efficacy of recombinant strains pPG-Aha1/Lc CC16 and pPG-Aha1-CTB/Lc CC16 via oral immunization, challenge tests were performed. On day 5 after the second booster immunization, each vaccinated fish was injected intraperitoneally with 200 μl of 5 × 10^6^ CFU (5LD_50_ dose) of *A. veronii*. Furthermore, the group of fish injected with 200 μl PBS was used as a blank control group. Each fish was monitored for 14 days after challenged and survival rate was analyzed in all the groups (n = 28 per group) post challenge.

### Statistical analysis

The statistical analysis of the data was performed by using SPSS v.16.0 software and GraphPad PRISM v7.0. The data was subjected to one way ANOVA followed by Tukey test to determine differences between groups. Mean values were considered significantly different when *p* < 0.05. Data are presented as mean ± standard deviation.

## Results

### Construction of recombinant *L. casei*

**Fig. 2:**
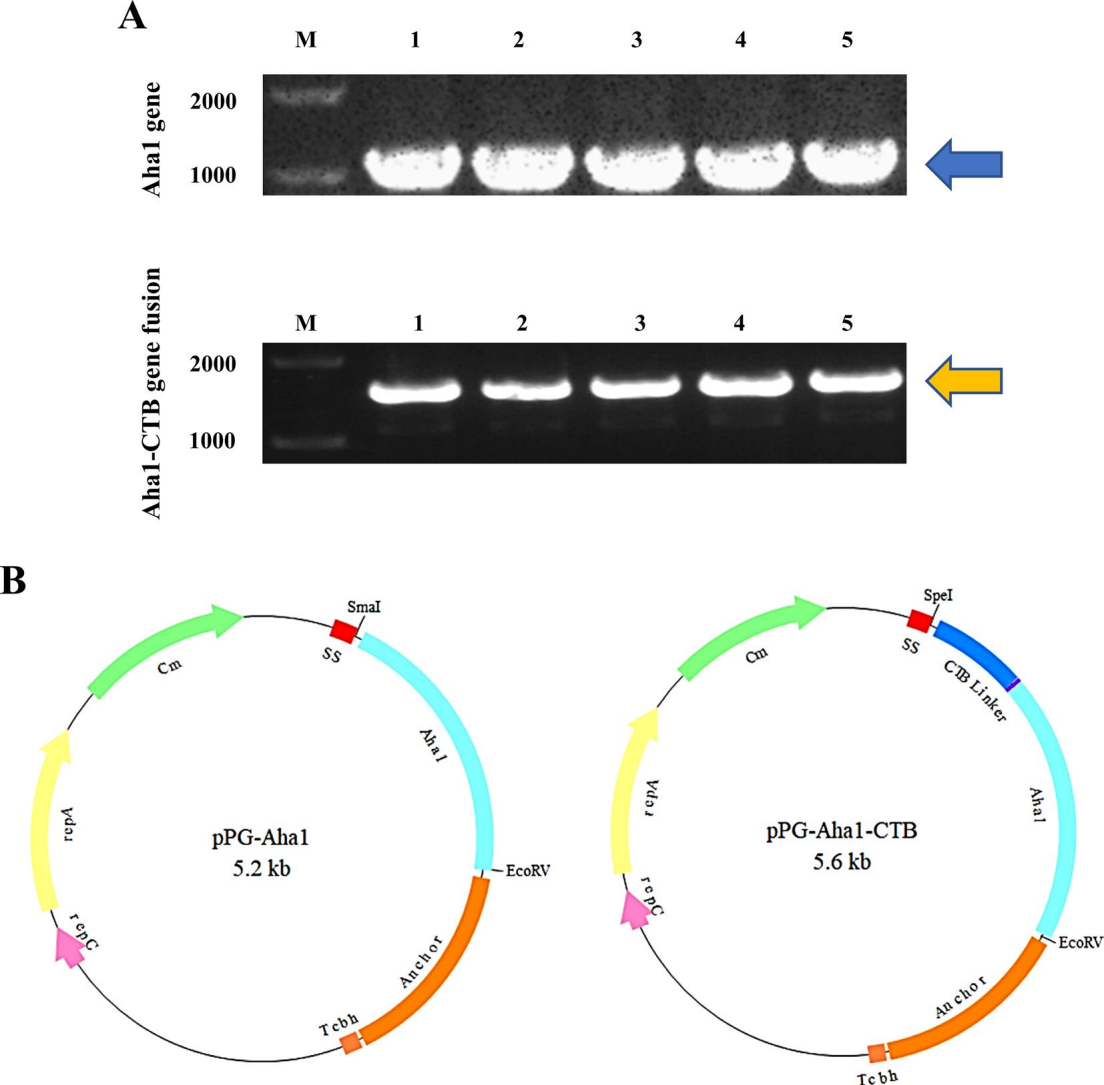
The identification of recombinant strains. Bands indicated by blue (1038 bp) or yellow (1449 bp) were analyzed by DNA sequencing and were consistent with the putative sequences, respectively. **A** The PCR amplification product of recombinant strains: M: DNA ladder (bp); Lane 1–5: PCR product of Cm-resistant clones. **B** Vector maps of the surface-displayed expressing, left: pPG-Aha1/Lc CC16; right: pPG-Aha1-CTB/Lc CC16

To verify the constructed recombinant plasmids, the recombinant expression plasmids pPG-Aha1 and pPG-Aha1-CTB were digested using *Sma*I/*Eco*RV or *Spe*I/*Eco*RV restriction enzymes (Takara, Japan), respectively. Meanwhile, the recombinants of *L. casei* confirmed by PCR (Fig. 2A) and sequence analysis. The sizes of target bands in electrophoretograms were the same as the designed sequence bands. And the recombinant strain was generated by electroporation of pPG-Aha1 and pPG-Aha1-CTB into *L. casei*, respectively (Fig. 2B). The sequence analysis indicated the successful construction of pPG-Aha1/Lc CC16 and pPG-Aha1-CTB/Lc CC16.

### Surface-displayed expression of recombinant *L. casei*

**Fig. 3:**
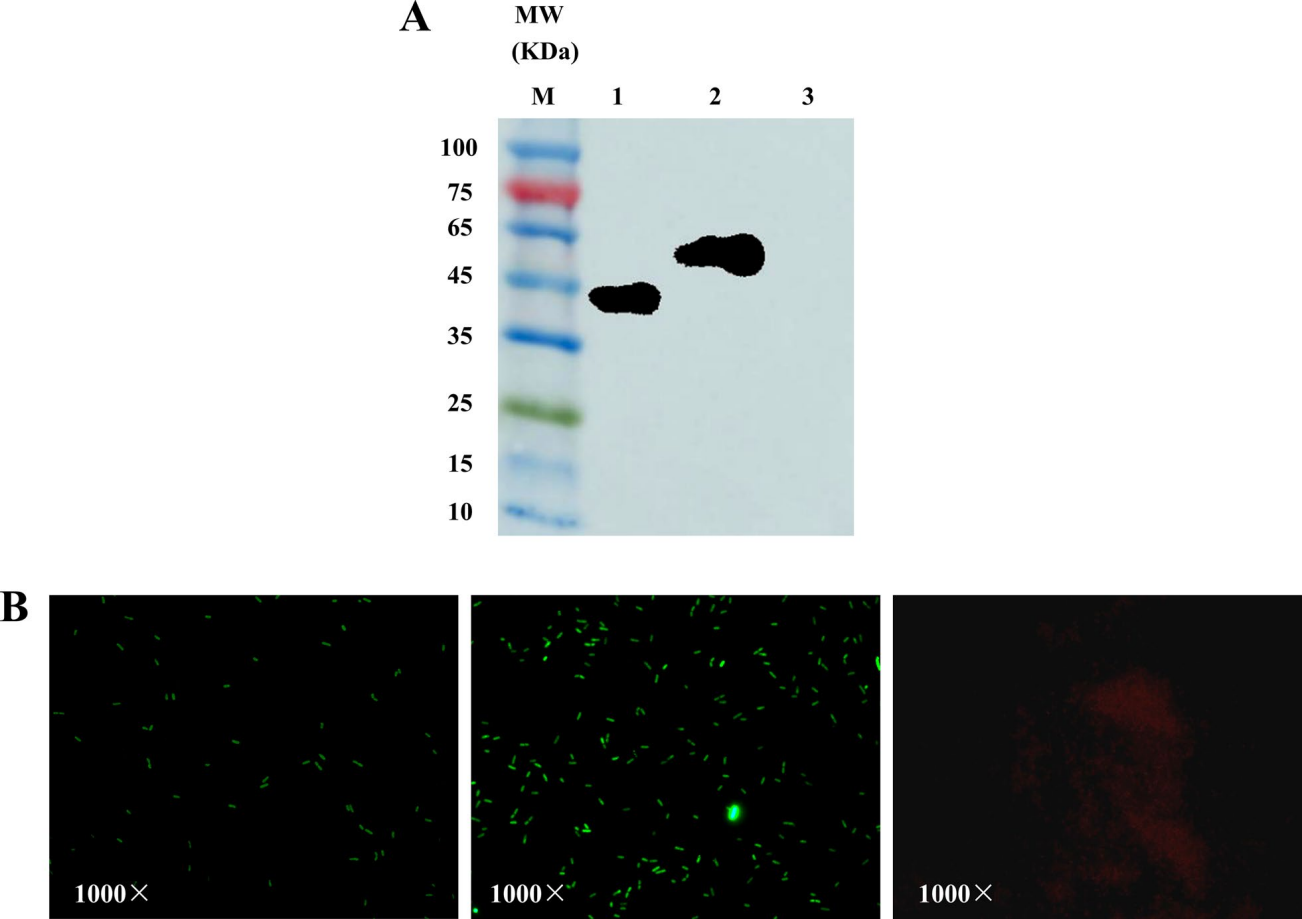
Expression of Aha1 protein from recombinant *L. casei* cell lysate by Western blot analysis and expression of Aha1 protein on recombinant cell surface by fluorescence microscopy analysis. **A** Identification of the proteins of expressed by the recombinant strains pPG-Aha1/Lc CC16 and pPG-Aha1-CTB/Lc CC16 by Western blotting with mouse anti-Aha1. MW indicates the molecular mass markers (kDa). 1: The expression of induced pPG-Aha1/Lc CC16; 2: The expression of induced pPG-Aha1-CTB/Lc CC16; 3: The expression of induced pPG/Lc CC16. **B** fluorescence microscopy analysis of Aha1 protein expression on recombinant cell surface. pPG-Aha1/Lc CC16 (left), pPG-Aha1-CTB/Lc CC16 (middle) and pPG/Lc CC16 (right), magnification: × 1000. There was green fluorescence on the surface of pPG-Aha1/Lc CC16 and pPG-Aha1-CTB/Lc CC16, respectively. As controls, no immunofluorescence reaction on the pPG/Lc CC16 cell surface

To investigate the expression of target protein, cell lysate from recombinant pPG-Aha1/Lc CC16, pPG-Aha1-CTB/Lc CC16 and pPG/Lc CC16 were subjected to SDS-PAGE followed by Western blotting analysis. Strong reactivity with anti-Aha1 serum antibody indicated affluent expression with the expected size of 44 kDa and 59 kDa, respectively, whereas pPG/Lc CC16 was not showing any reactive band (Fig. 3A). These results illustrated that Aha1 protein was successfully expressed.

Furthermore, for detecting the cellular localization of target protein on the surface of *L. casei*, Immunofluorescence assay was conducted which revealed fluorescent cells only in recombinant pPG-Aha1/Lc CC16 and pPG-Aha1-CTB/Lc CC16 but no green fluorescence emitted in recombinant pPG/Lc CC16, which were emitted red by Evans blue (Fig. 3B). The localization assay results propose that recombinant protein was anchored on the cell surface which maintained reactogenicity with the anti-Aha1 antibody.

### Genetic stability of recombinant *L. casei* strains

**Fig. 4:**
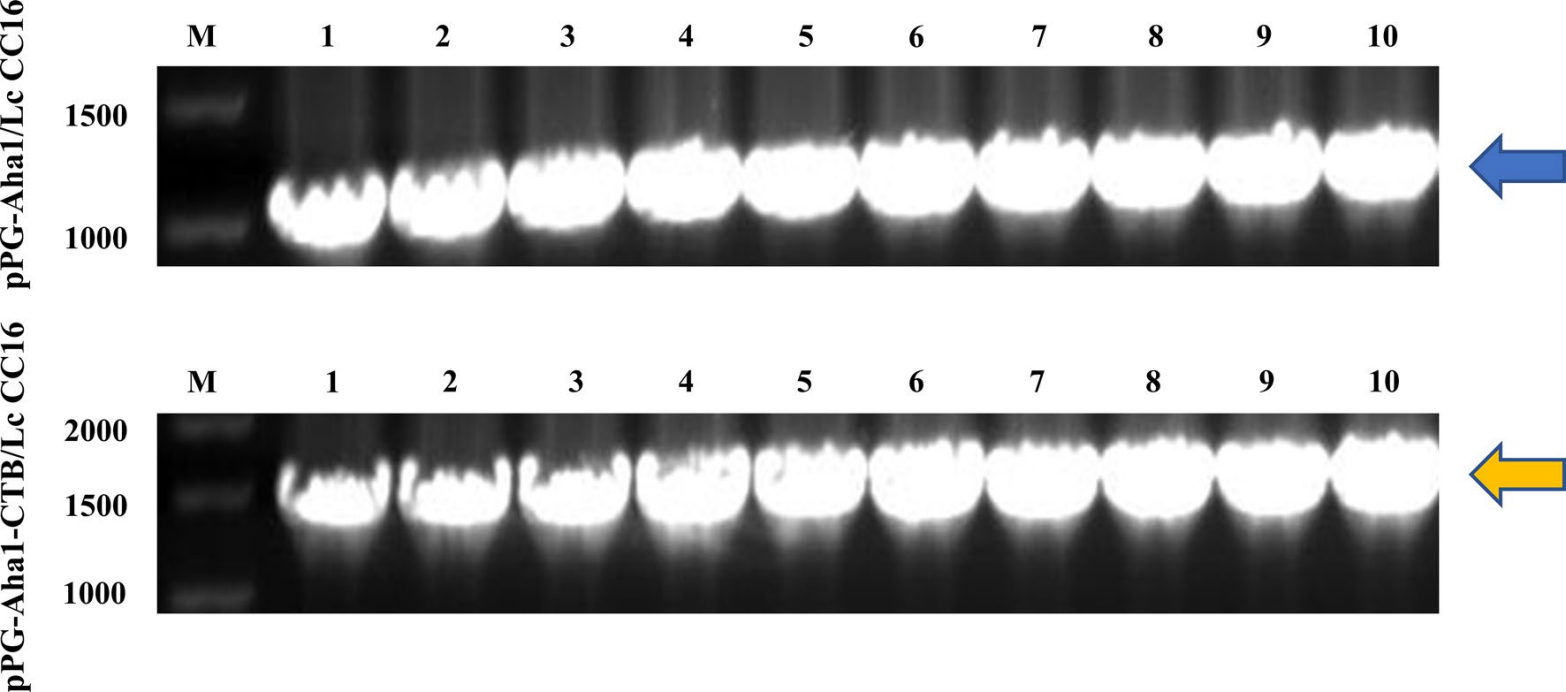
Detection of the genetic stability of recombinant *L. casei* strains. Bands indicated by blue (1038 bp) or yellow (1449 bp): The PCR analysis of recombinant pPG-Aha1/Lc CC16 plasmid or recombinant pPG-Aha1-CTB/Lc CC16 plasmid, respectively. M: DNA marker; Lanes 1–10: PCR amplification results of the 1 th to the 50 th generation of pPG-Aha1/Lc CC16 or pPG-Aha1-CTB/Lc CC16, respectively

To detect the genetic stability of recombinant pPG-Aha1/Lc CC16 and pPG-Aha1-CTB/Lc CC16, the recombinant *L. casei* strains were serially cultured for 50 generations and were screened by PCR and sequence analysis. As shown in Fig. 4, the sequence results were the same as the designed sequence, indicating good genetic stability.

### Humoral immune responses in *Cyprinus carpio* after vaccination

**Fig. 5:**
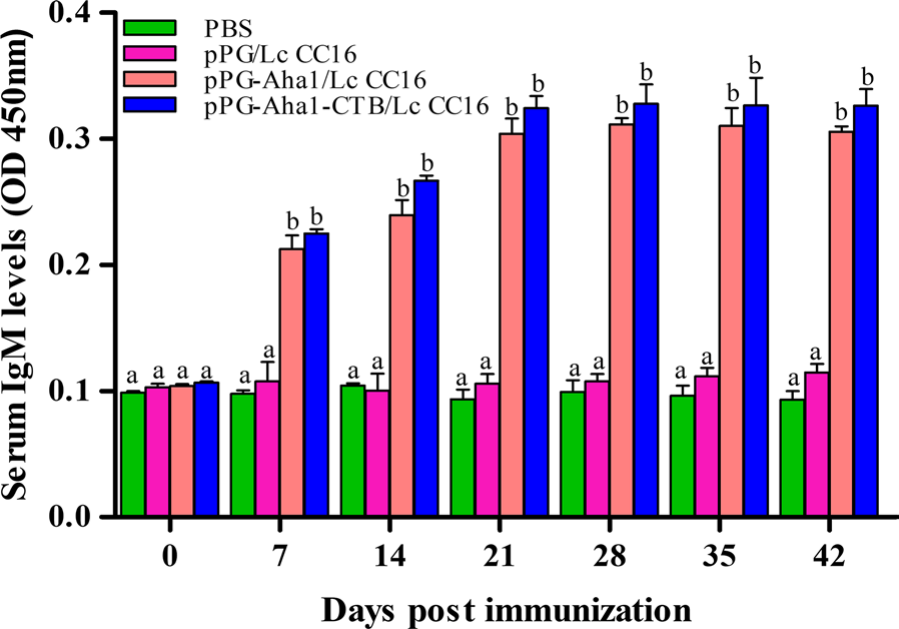
The levels of Aha1-specific IgM antibodies in the serum samples from *Cyprinus carpio* (n = 3 fish/group) immunized with pPG-Aha1/Lc CC16, pPG-Aha1-CTB/Lc CC16, pPG/Lc CC16 and PBS. Values are means for three assays and presented as the means ± SD as compared to PBS control. Dissimilar letters show significant difference (*p* < 0.05)

*Cyprinus carpio* were selected to evaluate the specific immunogenicity of generated vaccine candidate expressing Aha1 antigen through oral administration routes. Serum was analyzed for IgM (Fig. 5) consequently by ELISA using purified Aha1 protein as coating antigen. Two groups of *Cyprinus carpio* were immunized with the equal amount (10^9^ CFU/mL) of recombinant pPG-Aha1/Lc CC16 and pPG-Aha1-CTB/Lc CC16 through oral routes, respectively. Meanwhile, recombinant pPG/Lc CC16 and PBS as the control groups by oral administration were maintained. The results revealed that there were significant for IgM level among the vaccinated groups and control groups. Simultaneously, substantially higher IgM levels in the serum were detected in fish by oral routes of recombinant pPG-Aha1/Lc CC16 and pPG-Aha1-CTB/Lc CC16 compared to the control groups on day 28 (*p* < 0.05). On the contrary, there was no substantial differences in the two control groups.

**Fig. 6:**
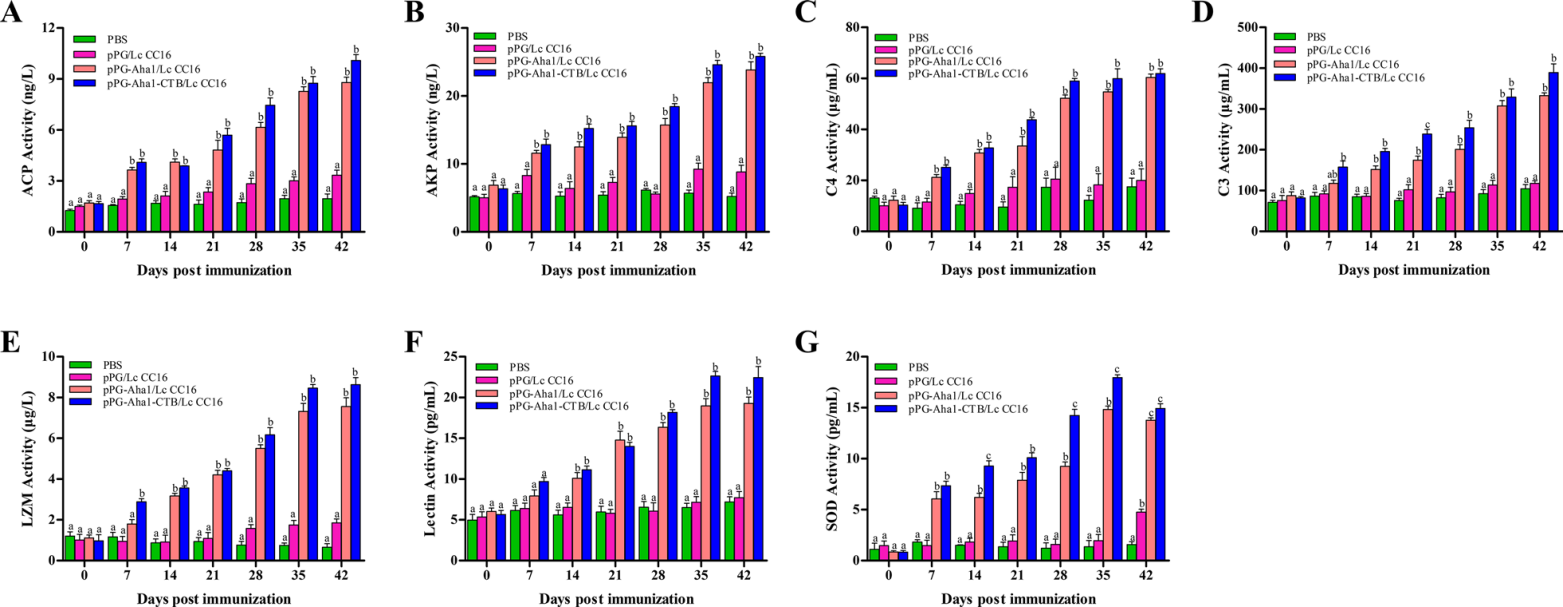
Changes of humoral immune parameters in the immunized *Cyprinus carpio* by recombinant *L. casei*. **A** ACP activity; **B** AKP activity; **C** C4 activity, **D** C3 activity, **E** LZM activity, **F** Lectin activity and **G** SOD activity. Date are represented as mean ± SE as compared to PBS control. Three fish per group were set for the tests. Dissimilar letters show significant difference (*p* < 0.05)

To detect the non-specific immune enzymes, ACP, AKP, LZM, C3, C4, SOD and Lectin activity were detected from serum. As the results showing, the ACP (Fig. 6A), AKP (Fig. 6B) and C4(Fig. 6C) activity in the serum were gradually raised after immunized recombinant pPG-Aha1/Lc CC16 and pPG-Aha1-CTB/Lc CC16 and induced the higher titers compared to the control groups on day 42 (*p* < 0.05). There was no substantial difference between recombinant pPG/Lc CC16 and PBS groups (*p* > 0.05). Meanwhile, C3 (Fig. 6D), LZM (Fig. 6E) and Lectin (Fig. 6F) activity in the serum were progressively increased by oral administration of recombinant pPG-Aha1/Lc CC16 and pPG-Aha1-CTB/Lc CC16 fish compared to controls after day 14 and peaked at day 42 (*p* < 0.05). However, the activity of C3 was significantly increased in the pPG-Aha1-CTB/Lc CC16 group compared to the pPG-Aha1/Lc CC16 group on day 21 and the LZM activity of pPG-Aha1-CTB/Lc CC16 was significantly higher than that of the other three groups on day 7 (*p* < 0.05). In contrast to the control groups, the result of SOD (Fig. 6G) activity in the serum were showed that recombinant pPG-Aha1/Lc CC16 and pPG-Aha1-CTB/Lc CC16 induced the higher titers on day 35 and a slight decrease on day 42 (*p* < 0.05). And the SOD activity of pPG-Aha1-CTB/Lc CC16 group was significantly higher than that of pPG-Aha1/Lc CC16 group at days 14, 28 and 35 (*p* < 0.05). By contrast, there was a difference between recombinant pPG/Lc CC16 and PBS groups only at day 42 (*p* < 0.05).

### Immune-related genes expression

**Fig. 7:**
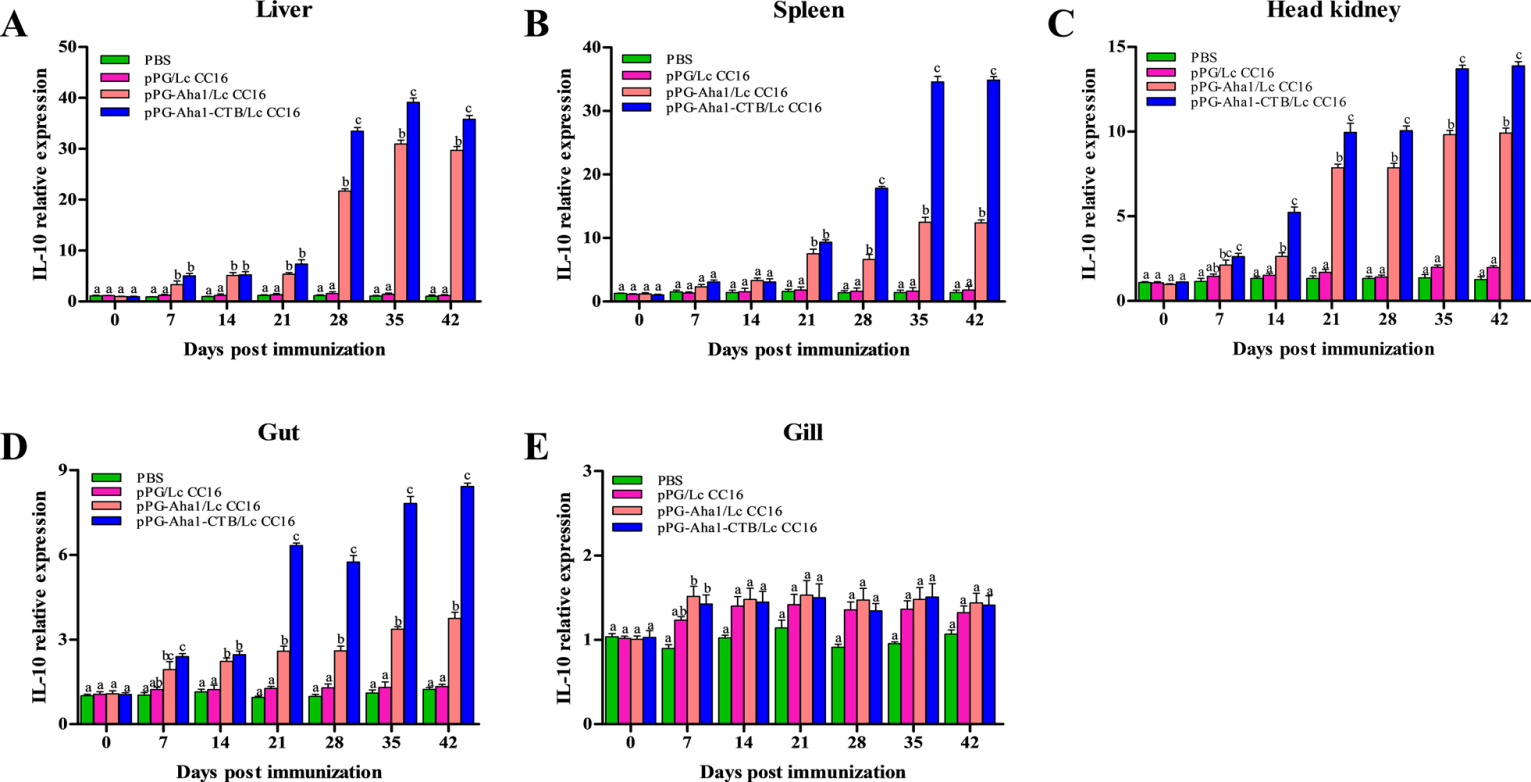
The SYBR Green real-time PCR analysis of the expression of IL-10 genes in liver (**A**), spleen (**B**), head kidney (**C**), intestine (**D**) and gill (**E**) of *Cyprinus carpio* (n = 3 fish/group) after immunized. Data are means for three assays and presented as the means ± SD fold increase relative to PBS. Dissimilar letters show significant difference (*p* < 0.05)

**Fig. 8:**
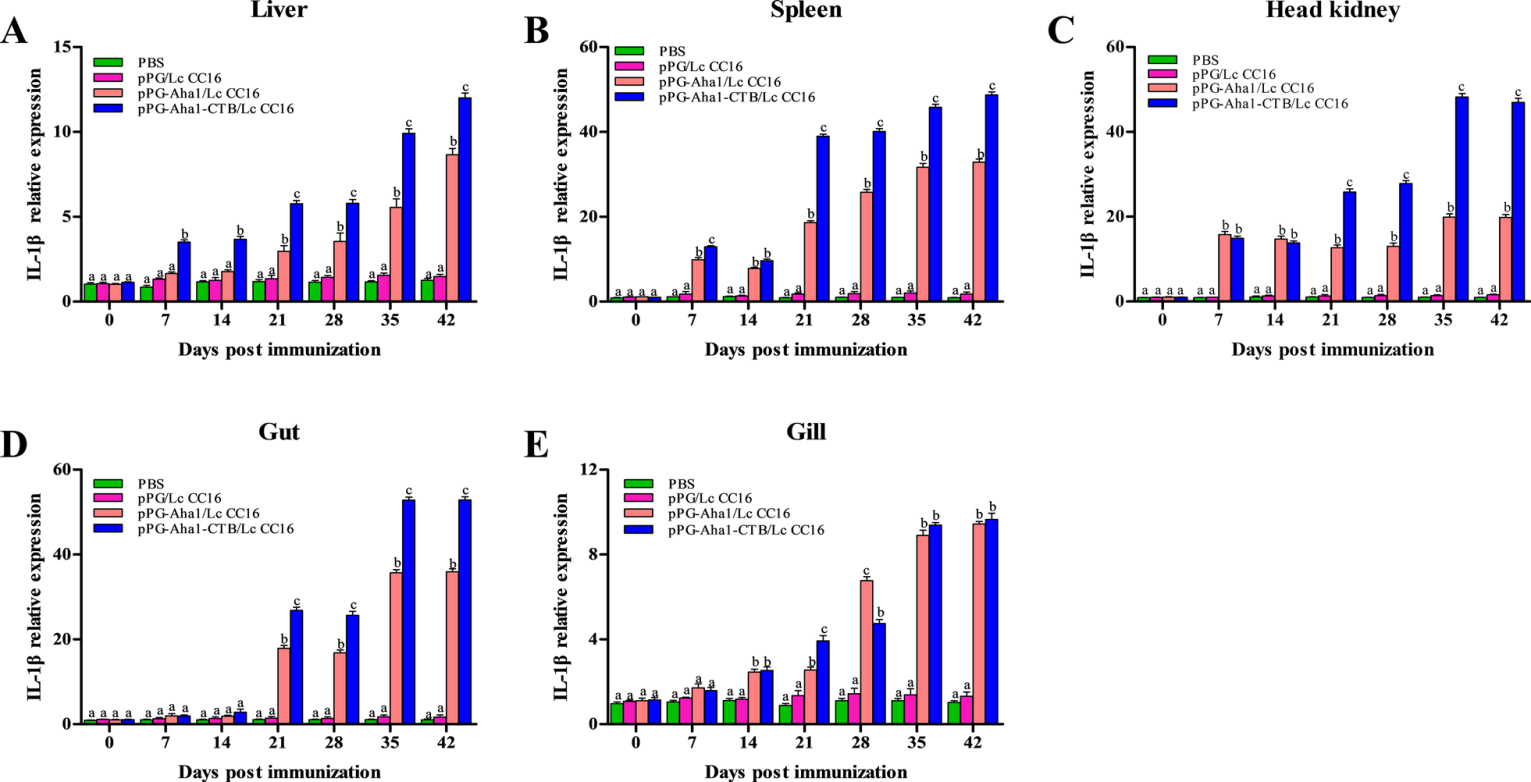
The SYBR Green real-time PCR analysis of the expression of IL-1β genes in liver (**A**), spleen (**B**), head kidney (**C**), intestine (**D**) and gill (**E**) of *Cyprinus carpio* (n = 3 fish/group) after immunized. Data are means for three assays and presented as the means ± SD fold increase relative to PBS. Dissimilar letters show significant difference (*p* < 0.05)

**Fig. 9:**
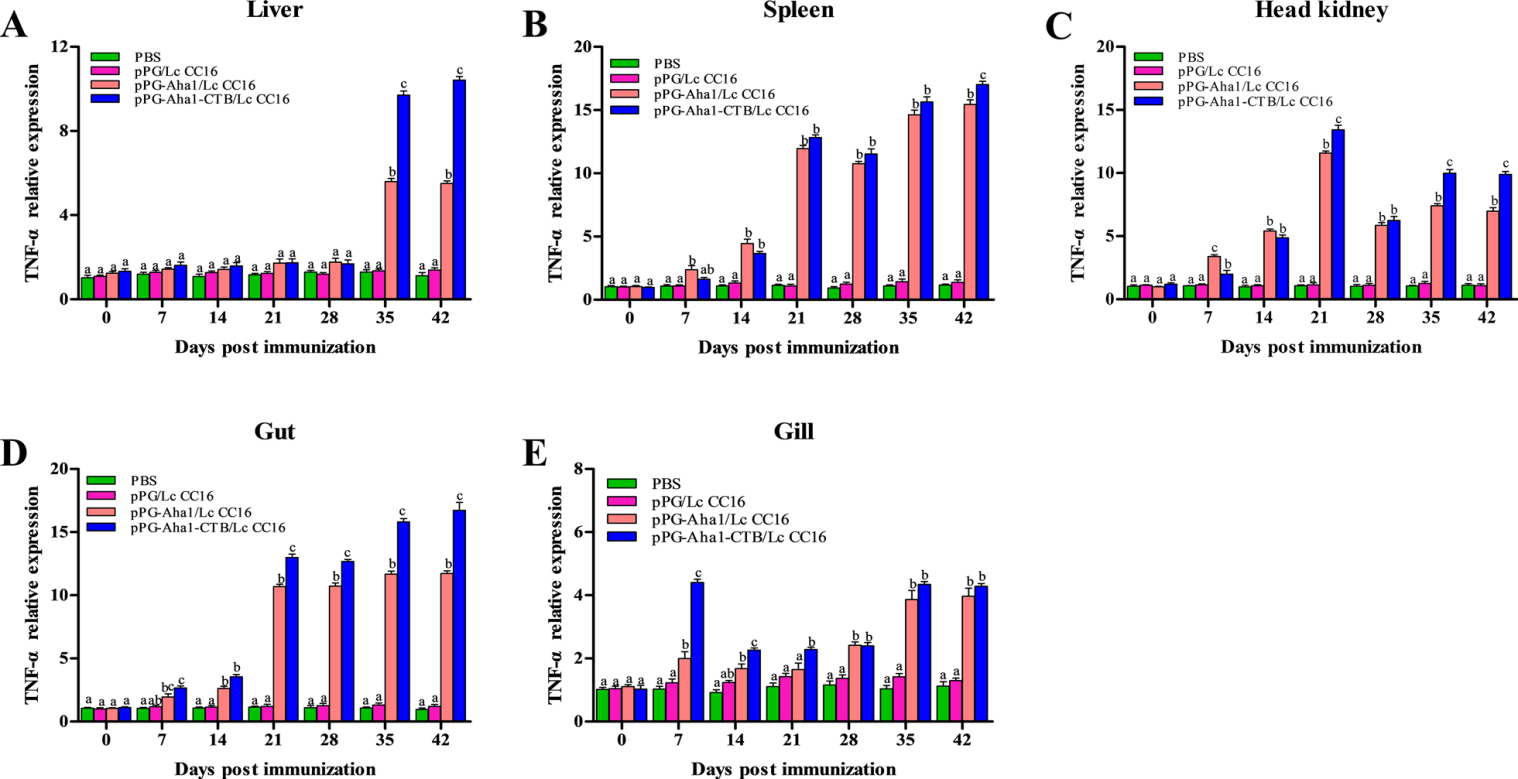
The SYBR Green real-time PCR analysis of the expression of TNF-α genes in liver (**A**), spleen (**B**), head kidney (**C**), intestine (**D**) and gill (**E**) of *Cyprinus carpio* (n = 3 fish/group) after immunized. Data are means for three assays and presented as the means ± SD fold increase relative to PBS. Dissimilar letters show significant difference (*p* < 0.05)

The effect of recombinant *L. casei* in the stimulation of immune responses were examined by measuring relative expression of IL-10, IL-1β, TNF-α, IgZ1 and IgZ2 by qRT-PCR analysis. In the pPG-Aha1/Lc CC16, pPG-Aha1-CTB/Lc CC16 groups, Fig. 7 shows that IL-10 gene expression was significantly rise in the liver, head kidney and gut after day 14 compared with the control groups (*p* < 0.05) and peaking at day 42. IL-10 expression in the spleen was rapidly increased after early booster immunization (at day 21). And the recombinant pPG-Aha1-CTB/Lc CC16 group showed superior results compared to the pPG-Aha1/Lc CC16 group. It is noteworthy that no obviously change in the gill in all groups (*p* > 0.05), except for day 7. IL-1β (Fig. 8) expression was significantly upregulation in different tissue in the pPG-Aha1/Lc CC16 and pPG-Aha1-CTB/Lc CC16 groups at early booster immunization (at day 21), peaking at the secondly booster immunization compared with the pPG/Lc CC16 and PBS (*p* < 0.05). This was notable, the pPG-Aha1-CTB/Lc CC16 group showed higher expression levels of IL-1β in the liver, spleen, head kidney and gut compared with the pPG-Aha1/Lc CC16 group (*p* < 0.05). However, IL-1β expression in the gill differed between the recombinant pPG-Aha1-CTB/Lc CC16 and pPG-Aha1/Lc CC16 groups only at days 21 and 28 (*p* < 0.05). As shown in Fig. 9, TNF-α expression in the spleen, head kidney and intestine of the recombinant pPG-Aha1/Lc CC16 and pPG-Aha1-CTB/Lc CC16 groups was increased rapidly at the early stage of immunization, with a decreasing trend at day 28, and reached the highest level at the second booster immunization (*p* < 0.05). The expression of TNF-α in the liver was observed to peak at the second booster immunization in the immunization group. Moreover, we observed that TNF-α expression in the gill of the pPG-Aha1-CTB/Lc CC16 was raised rapidly at the early stage of immunization, on the contrary, pPG-Aha1/Lc CC16 group reached the highest level at the secondly booster immunization (*p* < 0.05). Noticeably, pPG-Aha1-CTB/Lc CC16 group exhibited better effect compared to the pPG-Aha1/Lc CC16 group.

**Fig. 10:**
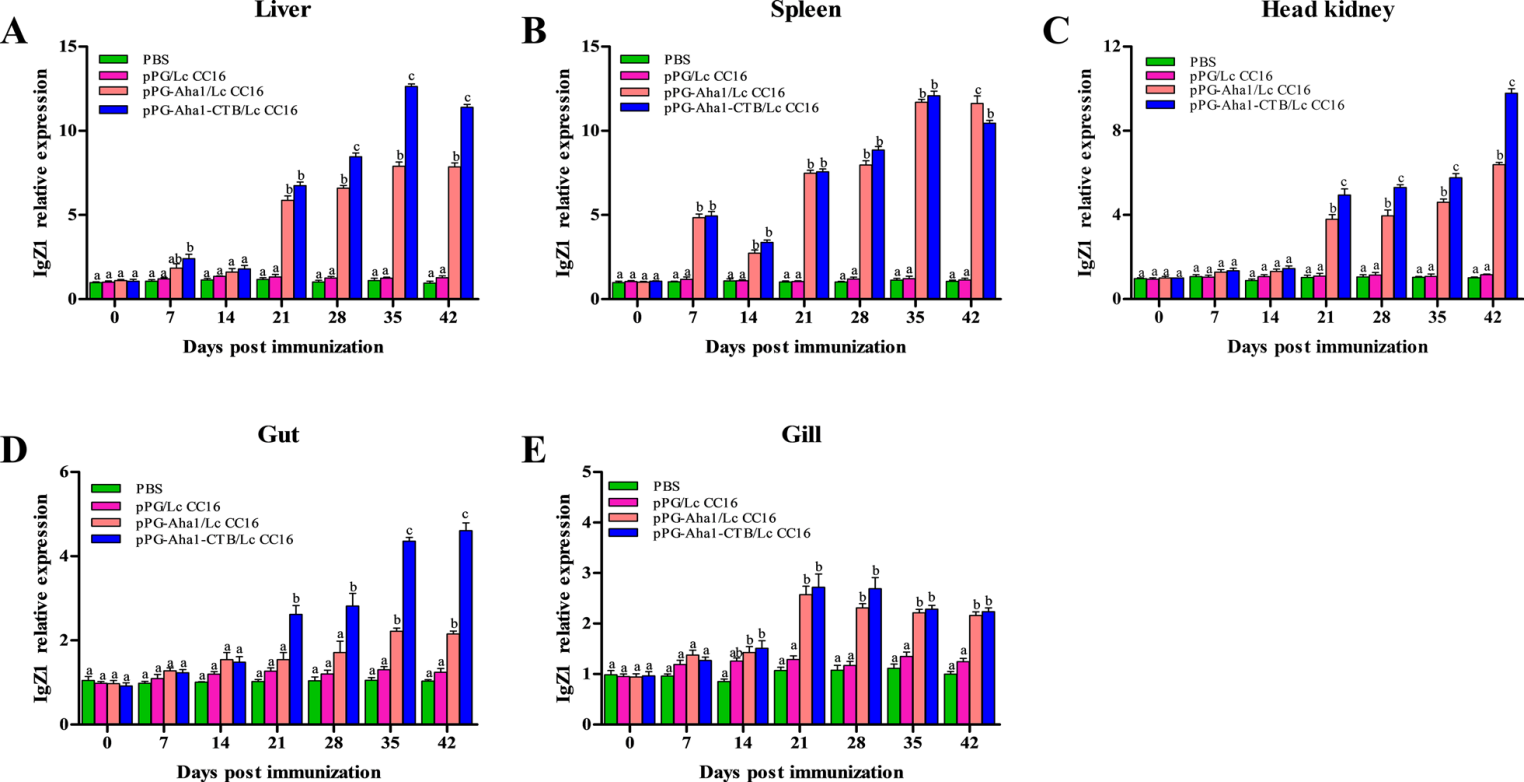
The SYBR Green real-time PCR analysis of the expression of IgZ1 genes in liver (**A**), spleen (**B**), head kidney (**C**), intestine (**D**) and gill (**E**) of *Cyprinus carpio* (n = 3 fish/group) after immunized. Data are means for three assays and presented as the means ± SD fold increase relative to PBS. Dissimilar letters show significant difference (*p* < 0.05)

**Fig. 11:**
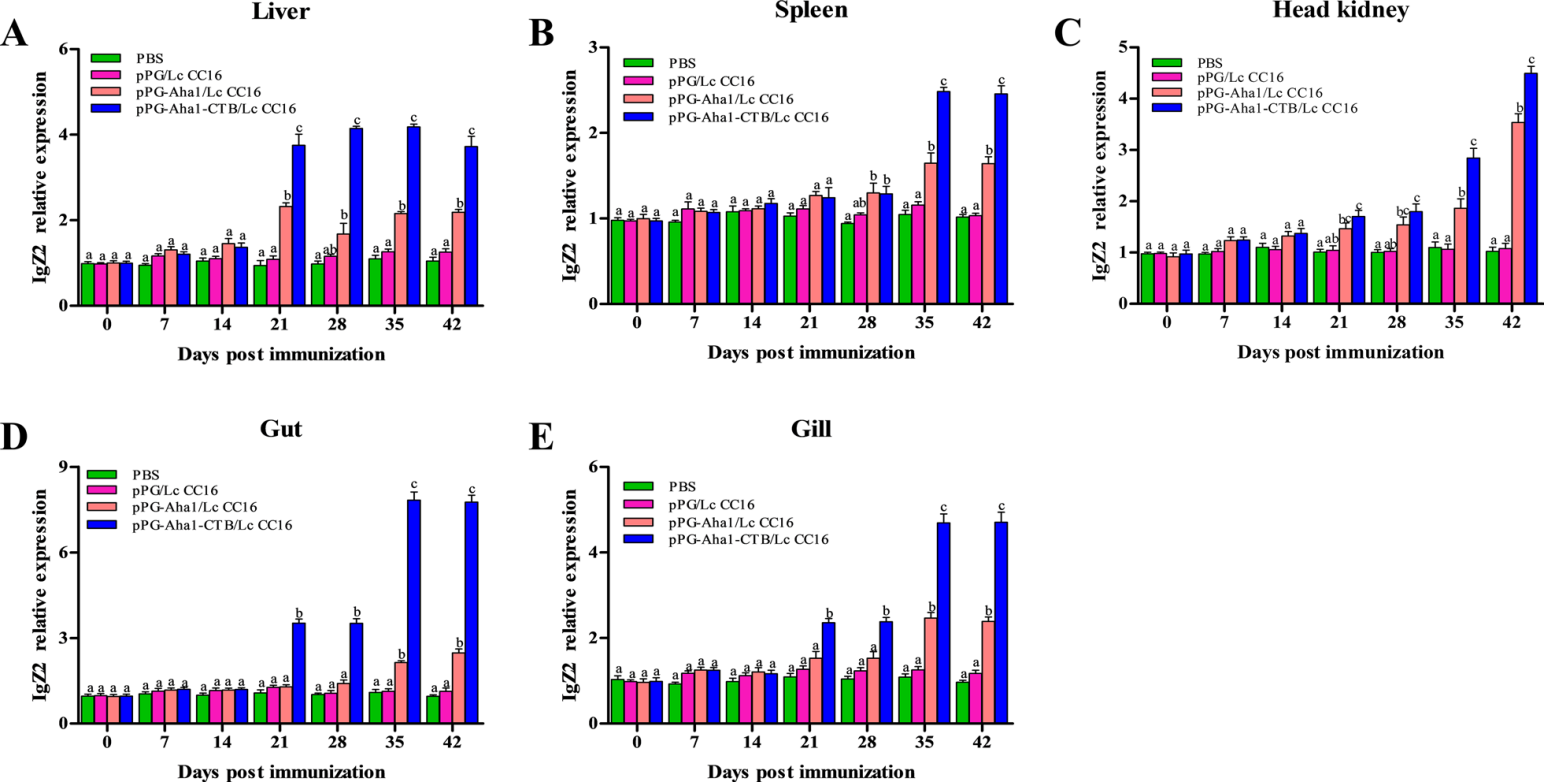
The SYBR Green real-time PCR analysis of the expression of IgZ2 genes in liver (**A**), spleen (**B**), head kidney (**C**), intestine (**D**) and gill (**E**) of *Cyprinus carpio* (n = 3 fish/group) after immunized. Data are means for three assays and presented as the means ± SD fold increase relative to PBS. Dissimilar letters show significant difference (*p* < 0.05)

After the early booster immunization, IgZ1 (Fig. 10) expression of the recombinant pPG-Aha1/Lc CC16 and pPG-Aha1-CTB/Lc CC16 groups were significantly increased in the liver, spleen, head kidney and gut, peaking at secondly booster and were significantly (*p* < 0.05) higher than the control groups. However, rapid change in the gill was observed in the pPG-Aha1/Lc CC16 and pPG-Aha1-CTB/Lc CC16 groups at day 21, and then showed a downward trend, while still higher than the pPG/Lc CC16 and PBS (*p* < 0.05). Of note, the expression of IgZ1 in the liver, kidney, and intestine of the pPG-Aha1-CTB/Lc CC16 group was significantly higher than the pPG-Aha1/Lc CC16 after boosted immunization (*p* < 0.05). Yet, there was no significant difference in the gill between the pPG-Aha1-CTB/Lc CC16 and pPG-Aha1/Lc CC16 groups (*p* > 0.05). Only at day 42, the level of IgZ1 in the spleen of the pPG-Aha1/Lc CC16 group was higher compared to pPG-Aha1-CTB/Lc CC16 (*p* < 0.05). In the pPG-Aha1/Lc CC16 and pPG-Aha1-CTB/Lc CC16 groups, IgZ2 (Fig. 11) expression was no obviously change in the spleen and head kidney at early immunization, but peaking at secondly booster immunization. Higher expression of IgZ2 in the liver was observed after day 21 (*p* < 0.05). In the gut and gill tissues, recombinant *L. casei* groups showed an increasing trend and reached the peak at the last booster immunization, which was significantly higher than the PBS group (*p* < 0.05). The result was notable that the IgZ2 expression of pPG-Aha1-CTB/Lc CC16 groups was significantly higher than that of Lc-pPG-Aha1 group.

### Colonization of recombinant *L. casei* in *Cyprinus carpio*

**Fig. 12:**
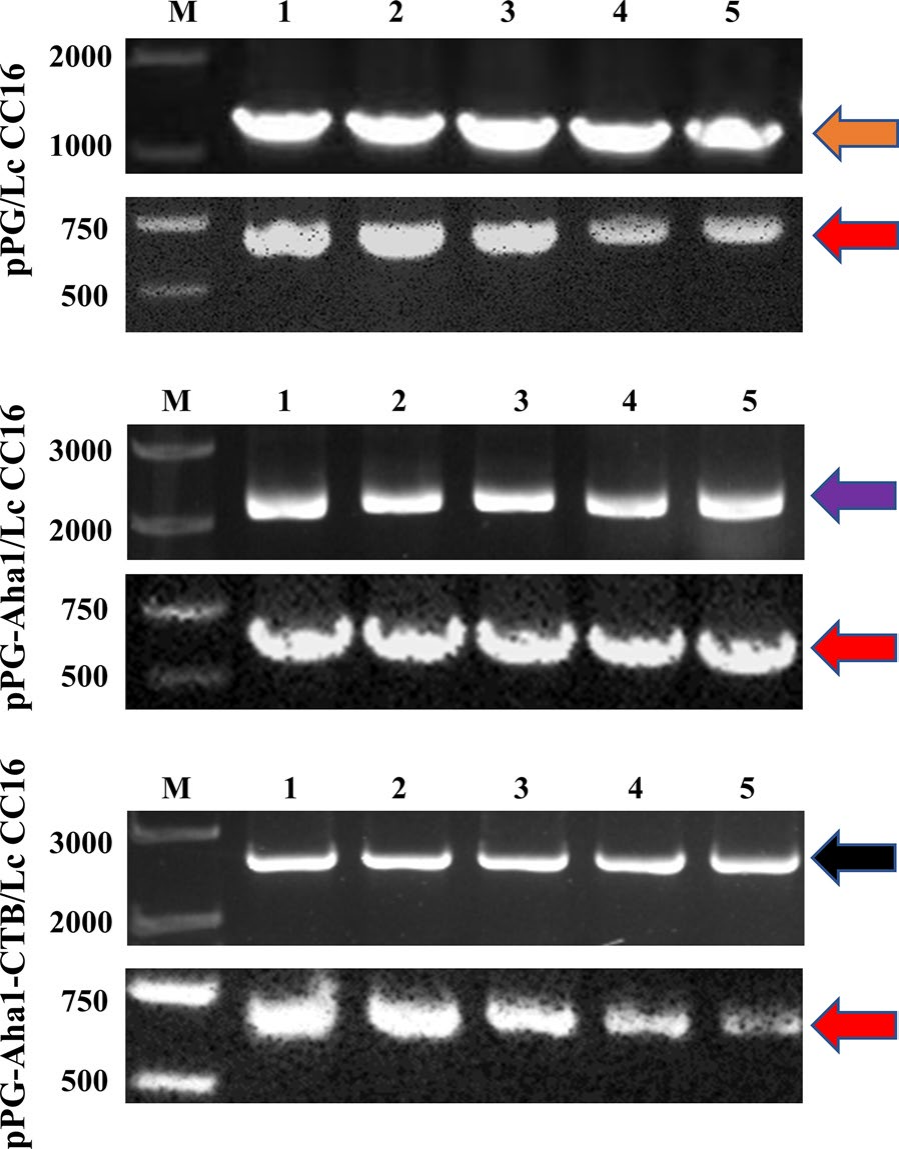
The identification of colonization ability of recombinant *L. casei* in the different intestinal sections of fish. The single colonies were randomly selected and conducted to PCR using pPG-vector specific primers (upper images of each group) and the housekeeping gene *dnaA* primers (lower images of each group). Bands indicated with orange (1250 bp), purple (2288 bp), black (2699 bp) and red arrows (615 bp) were further analyzed by DNA sequencing and were in concert with the putative sequences. M: DNA ladder (bp), Lane 1–5: PCR product of recombinant *L. casei* using specific primers

The colonization ability of recombinant pPG-Aha1/Lc CC16 and pPG-Aha1-CTB/Lc CC16 in the intestine was measured via observing the growth of the colony and using PCR-colony screening. Colony count results was shown in Table 2 and demonstrated that pPG-Aha1/Lc CC16 and pPG-Aha1-CTB/Lc CC16 could survive and colonize to the foregut, midgut and hindgut of fish. PCR results (Fig. 12) showed that a 615 bp PCR products was obtained using the housekeeping gene *dnaA*, and products of pPG/Lc CC16 (1250 bp), pPG-Aha1/Lc CC16 (2288 bp) and pPG-Aha1-CTB/Lc CC16 (2699 bp) were amplified with the pPG-specific primer pair, respectively. Of note, despite the high colonization rates in the midgut and hindgut, colonization was not consistent throughout each intestine segment.

**Table 2:** The colonization of recombinant *L. casei* in the intestine

Group/Days		Recombinant *L. casei* colony count(× 10^5^ CFU/mL)				
		1 d	3 d	7 d	12 d	18 d
Foregut	Lc-pPG-Aha1	4.63 ± 0.21^a^	4.83 ± 0.02^a^	3.66 ± 0.03^b^	1.35 ± 0.13^c^	0.87 ± 0.03^c^
	Lc-pPG-Aha1-CTB	4.42 ± 0.13^a^	4.85 ± 0.02^a^	4.13 ± 0.21^b^	1.72 ± 0.07^c^	1.16 ± 0.1^c^
	Lc-pPG	2.6 ± 0.12^a^	2.72 ± 0.01^a^	2.12 ± 0.11^b^	1.4 ± 0.14^c^	0.85 ± 0.03^d^
	Control	–	–	–	–	–
Midgut	Lc-pPG-Aha1	4.19 ± 0.14^a^	5.34 ± 0.13^b^	5.57 ± 0.01^b^	5.01 ± 0.18^b^	5.28 ± 0.11^b^
	Lc-pPG-Aha1-CTB	5.8 ± 0.13^a^	6.11 ± 0.1^a^	6.24 ± 0.02^a^	5.55 ± 0.12^a^	5.66 ± 0.19^a^
	Lc-pPG	1.61 ± 0.12^a^	2.14 ± 0.13^a^	1.81 ± 0.12^a^	0.83 ± 0.02^b^	0.97 ± 0.02^b^
	Control	–	–	–	–	–
Hindgut	Lc-pPG-Aha1	4.82 ± 0.11^a^	4.86 ± 0.03^a^	6.93 ± 0.29^b^	5.75 ± 0.04^c^	6.07 ± 0.2^c^
	Lc-pPG-Aha1-CTB	7.16 ± 0.05^a^	7.76 ± 0.13^a^	8.54 ± 0.21^a^	7.24 ± 0.03^b^	7.68 ± 0.21^b^
	Lc-pPG	1.49 ± 0.14^a^	2.24 ± 0.03^b^	1.94 ± 0.01^b^	1.2 ± 0.03^c^	1.13 ± 0.01^c^
	Control	–	–	–	–	–

Data are means for three assays and presented as the means ± SD fold increase relative to PBS. Three fish per group were set for the tests. Dissimilar letters show significant difference (*p* < 0.05)

### Protection against *A. veronii* challenge

**Fig. 13:**
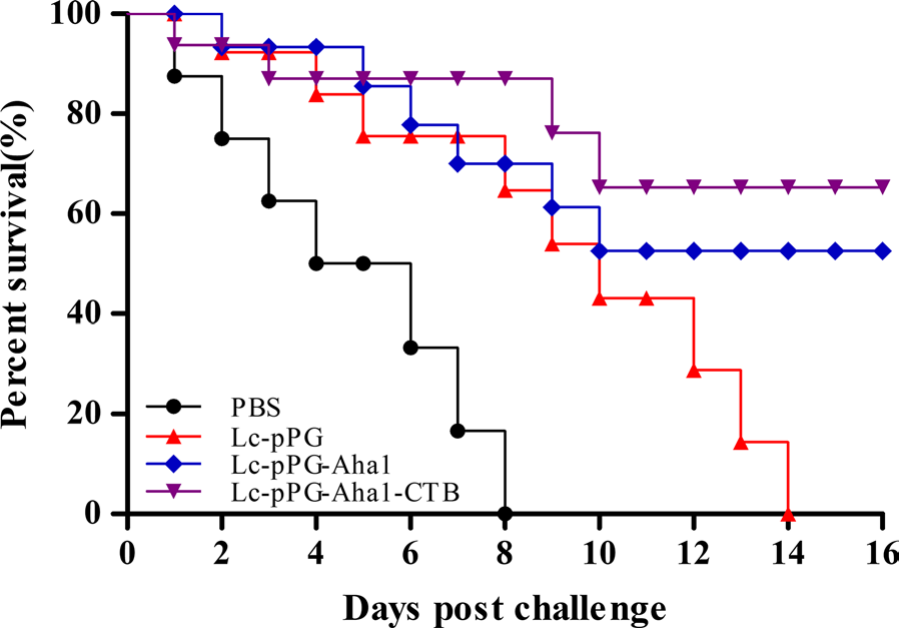
Immune protection efficacy of recombinant *L. casei*. Survival rate of *Cyprinus carpio* orally immunized with pPG-Aha1/Lc CC16, pPG-Aha1-CTB/Lc CC16, pPG/Lc CC16 and PBS following challenge with *A. veronii* TH0426 after immunization. 28 fish/group were used for documenting the percentage of survival for 14 days

The protective immunity of the recombinant *L. casei* was evaluated via challenge with a lethal dose of *A. veronii* TH0426. As shown in Fig. 13, analysis revealed the higher survival of fish immunized with recombinant pPG-Aha1/Lc CC16 and pPG-Aha1-CTB/Lc CC16 when compared with that of the control groups and pPG/Lc CC16. At the end of the challenge, *Cyprinus carpio* immunized with recombinant pPG-Aha1/Lc CC16 and pPG-Aha1-CTB/Lc CC16 exhibited survival rates of 53.57% and 64.29%, respectively, while all fish from the control groups died.

## Discussion

*A. veronii* is an important human, animal and aquatic organism pathogen, which can cause high mortality of *Cyprinus carpio* and other aquatic organisms. The extensive use of antibacterial drugs in the aquaculture enhances the rate of antimicrobial resistance evolution. In this scenario, seeking new and effective solutions is crucial for the aquaculture industry.

In recently, vaccination is usually carried out by injection, immersion and oral administration in the aquaculture industry. And the oral immunization has many advantages, such as convenient administration and suitable for mass immunization, which has been studied extensively in recent years [41]. For effectively delivering the vaccine orally, antigens must avoid the enzymatic degradation of the digestive tract and promote the identification and uptake of the antigen in the intestine to induce an effective protective immune response. Meanwhile, the subunit vaccine is easily degraded by the digestive tract, juice, and enzymes, which not deliver to the presentation of drug antigen therapy [42]. Therefore, it is very important to choose a suitable antigen deliver carrier to protect the antigen from being degraded. In this study, the recombinant *L. casei* expressing Aha1 protein of *A. veronii* with xylose-induced expression systems in conjunction with CTB as mucosal adjuvant was constructed and the immunogenicity and induction of immunity response using *Cyprinus carpio* as an animal model was evaluated.

Up to now, a series of heterologous antigen have successfully expressed in *L. casei*, such as OmpA (surface-displayed), OmpW (surface-displayed) and Malt (surface-displayed) [28, 43, 44]. As a deliver carrier, *L. casei* was used very proficiently whereas desired result was also found when it was used as a carrier in DNA vaccine through oral immunization in fish. In our study, the recombinant pPG-Aha1/Lc CC16 and pPG-Aha1-CTB/Lc CC16 were passed for at least 50 generations and showed good genetic stability with no gene deletion. In the previous report, thymidine auxotrophic (ΔthyA) recombinant *L. casei* expressing bovine lactoferricin [45] was constructed which showed similar genetic stability to this study. These data provided a possible explanation that recombinant strains have great colonize ability, which is conducive to the continuous expression and secretion of protein, and stimulates the local mucosal immune response of body.

The humoral immunity of fish is mainly composed of specific immunity and non-specific immunity. Among them, specific immunity is mainly mediated by IgM, as an important immune parameter in serum of immunized fish, which can defend against infections such as bacteria, toxins and viruses in the blood [46]. In this study, we compared two recombinant *L. casei* (surface-displayed) to induce fish body-specific IgM antibody levels after immunized carp, and the results showed that significant levels of IgM antibody were induced in the fish immunized with recombinant *L. casei* than other two groups. However, pPG-Aha1-CTB/Lc CC16 induced IgM antibody levels slightly higher than pPG-Aha1/Lc CC16. We hypothesized that the Aha1 protein expressed by recombinant *L. casei* reached to intestine mucosal tissues and elicited local mucosal immune response, and IgM was released into blood subsequently stimulating systemic immune response which increasing the contact of the surface antigen with epithelial cells and immune cells, thereby promoting the differentiation of Ig^+^B cells, and the addition of mucosal immune adjuvants to enhance the level of immune response. Our results are in accordance with the results of a previous report that significant IgM antibody levels were detected in fish immunized with recombinant *L. casei* displayed AHA1-CK6 and VP2, and AHA1 as vaccine adjuvant could enhance the levels of IgM [47].

The non-specific immunity of fish mainly products in the blood and mucous membranes, including different types of cells (leukocytes and macrophages) and their products (complement, lysozyme, superoxide dismutase, etc.), which can resist pathogen infection. AKP and ACP are two important hydrolytic enzymes, which are involved in signal transduction and energy conversion, which regulate non-specific immunity and nutrient metabolism. In previous studies, Tian et al. [39] reported that significant increased activity of serum AKP and ACP in fish after oral immunization of crucian carp recombinant *L. casei*. Similarly, our data showed that the activity of serum AKP and ACP were enhanced in the immune fish. Our findings demonstrated that recombinant *L. casei* may improve innate immune response. In fish, C3 and C4 are an acute phase reactive protein, as one of the main components of the complement system, play an important role in the defense process of natural immunity. Reports showed that the activity of C3 and C4 were significant higher after boosted immunization. And the similar report that Shabot was fed with *L. casei* [48]. Our findings demonstrated that recombinant *L. casei* was able to stimulate complement receptor expression. LZM is a non-specific defense protein that exists in fish, an important indicator of immune function and body condition, play potential roles in fighting fish infections. In the present study, the enhancement of LZM activity was observed, in which the similar findings in *Cyprinus carpio* fed with recombinant *L. casei* [49]. These investigators commented that recombinant *L. casei* could enhance the serum LZM activity of the non-specific immune system of common carp. Lectin is the main non-specific immune factor and antibacterial related factor of fish, which can participate in the immune response and prevent the invasion of pathogenic bacteria. we conjectured that the activity of serum Lectin was enhanced in the immune fish, in which may be decreased antigen stimulation, leading to a dynamic equilibrium state of activity. SOD is an antioxidant defense enzyme that can be used to assess non-specific immune responses and also plays an important role in resisting pathogen invasion. Our results revealed that the activity of SOD gradually increased and reached the highest level at day 35, and finally decreased. We hypothesized that that recombinant *L. casei* play potential roles in boosting antioxidant capacity of fish. This result was consistent with studies in which *Takifugu obscurus* fed with Isatis root polysaccharide and sea cucumber *Apostichopus japonicus* fed with probiotics [50, 51]. It is interested to highlight that pPG-Aha1-CTB/Lc CC16 can induce higher levels of these enzymes than pPG-Aha1/Lc CC16, indicating CTB as an immunoadjuvant is capable of efficiently fulfilling a role in immune defense.

Cytokines are pleiotropic polypeptides that control cell development and immune cell-mediated responses, in which play a major role in host innate immunity. And are also recruited and activated by macrophages, neutrophils, and macrophages that Eliminate pathogen infections. IL-10 is considered to be an anti-inflammatory cytokine produced by various different types of immune cells and tissue epithelial cells, which can inhibit the intensification of inflammatory response when oneself cells and tissues are damaged. This study reveal that the expression of IL-10 was significantly rise in the liver, spleen, head kidney and gut on day 14 compared with the control groups. The similar phenomenon was observed that increase the expression of IL-10 gene after oral administration of immune *Lactococcus lactis* or *Lactobacillus plantarum* in *Paralichthys olivaceus* [52]. And we conjectured the production of IL-10 by engineered *L. casei* could alleviate the inflammatory response and reduce body damage. IL-1β and TNF-α are both pro-inflammatory cytokines, mainly produced by activated macrophages, in which play a determinant in initiating the pre-inflammatory cytokine cascade, macrophage recruitment and activation, and stimulating adaptive immune responses. The recombinant *L. casei* groups showed obviously enhance IL-1β and TNF-α expression in different tissues compared with the control groups. Similar to our results was observed in the enhancement of IL-1β and TNF-α expression in *Oreochromis niloticus* fed with probiotics [53]. It is possible that up-regulated IL-1β and TNF-α might trigger early inflammatory response, enhance immune response, which lead to up-regulation of pro-inflammatory cytokine levels. Immunoglobulins (Igs) are a type of globulin produced by B cells that can specifically recognize and neutralize corresponding antibodies. At present, there are three subtypes of mucosal immunoglobulin in carp, which IgZ1 is expressed systemically, IgZ2 is preferentially expressed in mucosal sites, and IgT is expressed in head kidney, gill and blood cells. We noticed that recombinant *L. casei* groups displayed a higher upregulation of IgZ1 in the liver, spleen and head kidney, and a significant upregulation of IgZ2 in the gut and gill. Similarly, the study conducted by Ryo et al. illustrated that IgZ1 is more abundant in systemic organs and IgZ2 chimera is preferentially expressed at mucosal sites in carps [54]. This suggests IgZ1, the function in neutralization of antigens in the blood, was expressed systemically and IgZ2 shows an important in protecting the host from *A. veronii* infection. It has become interesting that pPG-Aha1-CTB/Lc CC16 can enhance robust the levels of cytokine, indicating that the fusion of immunoadjuvant is effective in inducing cellular immune responses.

For a mucosal vaccine, the colonization ability in the intestinal tract is important. After oral immunization, the colonization of the recombinant pPG-Aha1/Lc CC16 and pPG-Aha1-CTB/Lc CC16 were detected, and the data showed recombinant *L. casei* could survive in carp intestine and the colonization ability of midgut and hindgut is stronger than foregut. We surmised that the colonization of the recombinant *L. casei* was gradually decreased, which may be the metabolism of carp and the effects of the digestive tract environments. Similarly, the study investigated by Duan et al. demonstrated that recombinant Lactobacillus expressing CK6 fused with VP2 protein could survive and attach to the surface of intestinal tract of Rainbow trout [55]. And our data exhibited that the recombinant *L. casei* can survive to the intestinal tract in fish.

In the study, our data pointed that recombinant *L. casei* can resist the *A. veronii* infection, which have a certain protective effect. Notably, the immune protection effect of pPG-Aha1-CTB/Lc CC16 group (64.29%) is stronger than that of pPG-Aha1/Lc CC16 group (53.57%), which may be the inclusion of mucosal immune adjuvant slows down the damage caused by the inflammatory response of the intestinal mucosa and enhances immunity in *Cyprinus carpio*. In further research, testing the immunization doses and immunization times of recombinant *L. casei* that enhancing the immunity of the vaccine are needs to be explored. And it is necessary to explore the intestinal flora composition and function of *Cyprinus carpio* immunized with recombinant *L. casei*.

## Conclusion

In conclusion, the recombinant *L. casei* vaccine can effectively stimulate *Cyprinus carpio* to produce humoral and cellular immunity, which can be colonized in the intestine, and provide immune protection by oral route. And the combination of recombinant *L. casei* expressing Aha1 with the immune adjuvant CTB also enhanced the immune response of *Cyprinus carpio*. Collectively, our study provides a promising strategy for vaccine development against *A. veronii* infection in *Cyprinus carpio*.

## Supplementary Information

**Additional file 1: Table S1**. Information on the weight and length of all the healthy Cyprinus carpio (n=70 per group).
