# Supplementary material for: Oral vaccination with recombinant Lactobacillus casei expressing Aha1 fused with CTB as an adjuvant against Aeromonas veronii in common carp (Cyprinus carpio)
Source: Microb Cell Fact. 2022 Jun 13;21:114. doi: 10.1186/s12934-022-01839-9 (PMC9191526; doi:10.1186/s12934-022-01839-9)
Supplement: Supplementary file 1 — Additional file 1: Table S1. Information on the weight and length of all the healthy Cyprinus carpio (n=70 per group). [file 12934_2022_1839_MOESM1_ESM.pdf]

Oral vaccination with recombinant *Lactobacillus casei* expressing Aha1 fused with CTB as an adjuvant against *Aeromonas veronii* in common carp (*Cyprinus carpio*)

Chong Chen[a†], Shuo Zu[b†], Dongxing Zhang[c], Zelin Zhao[c], Yalu Ji[a], Hengyu Xi[a], Xiaofeng Shan[c], Aidong Qian[c]*, Wenyu Han[a, d]*, and Jingmin Gu[a, d*]

a. State Key Laboratory for Zoonotic Diseases, Key Laboratory of Zoonosis Research, Ministry of Education, College of Veterinary Medicine, Jilin University, Changchun 130062, People's Republic of China

b. Key Laboratory of Bionic Engineering, Ministry of Education, Jilin University, Changchun 130025, People's Republic of China

c. College of Animal Science and Technology, Jilin Agricultural University, Changchun 130118, People's Republic of China

d. Jiangsu Co-Innovation Center for the Prevention and Control of Important Animal Infectious Diseases and Zoonoses, Yangzhou University, Yangzhou 225009, People's Republic of China

*Corresponding author: Aidong Qian (qianaidong0115@163.com), Wenyu Han (hanwy@jlu.edu.cn) and Jingmin Gu (jingmin0629@163.com)

† Chong Chen and Shuo Zu contributed equally to this article.

Supplementary Information

Additional file 1: Table S1. Information on the weight and length of all the healthy *Cyprinus carpio* (n=70 per group).

Number/Groups	PBS		pPG/Lc CC16		pPG-Aha1/Lc CC16		pPG-Aha1-CTB/Lc CC16	
	Weight (g)	Length (cm)	Weight (g)	Length (cm)	Weight (g)	Length (cm)	Weight (g)	Length (cm)
1	59.51	14	60.7	13.89	59	13.71	60.85	13.68
2	60.12	13.98	60.59	13.97	60.12	13.78	60.74	13.59
3	59.23	13.52	59.89	13.81	60.17	13.96	60.38	13.68
4	59	13.76	59.9	13.65	60.25	13.92	60.92	13.87
5	60.57	13.88	60.7	13.78	60.75	13.94	61	13.97
6	61	13.65	60.58	13.67	60.34	14.01	61	13.65
7	60.52	13.78	59.85	13.51	60.12	13.89	59	13.58
8	60.81	13.61	59.62	13.59	60.45	13.55	59.5	13.62
9	60.37	13.55	59.78	13.6	59.98	13.65	60.34	13.9
10	59.3	13.6	60.21	13.53	59.8	13.81	60.2	13.65
11	60.75	13.76	60.78	13.7	60.1	13.87	60	13.58
12	60.58	13.88	60.65	14	60.78	13.79	59.21	14
13	60.34	13.99	60.12	14	60.99	13.62	60	13.98
14	59.07	13.65	60.5	13.89	60.45	13.84	59.78	13.51
15	59.3	13.53	61	13.78	60.7	13.73	59.63	13.61
16	59.56	13.51	61	13.79	59.78	13.62	59.78	13.66
17	59.62	13.7	59.45	13.55	59.2	13.58	60.25	13.52
18	59.55	13.55	59.62	13.61	59.35	13.9	60.98	13.71
19	59.62	13.89	59.78	13.58	59.12	13.56	61	13.99

20	59.78	13.95	59.6	13.88	59.46	13.66	60.78	13.68
21	59.32	13.76	59.21	13.51	59.43	13.74	60.35	13.62
22	59.6	13.58	60.75	13.71	60.1	13.68	59.49	13.78
23	60.25	13.63	59.23	13.76	59.8	13.61	59.38	13.78
24	60.35	13.7	59.6	13.57	60.12	13.59	59.66	13.65
25	59.85	13.59	59.62	13.61	59.74	13.77	60.11	13.77
26	59.63	13.68	60.12	13.72	59.63	13.58	60.45	13.7
27	59.78	13.65	60.45	13.73	59.74	13.99	60.5	13.69
28	60.26	13.88	60.78	13.69	60.21	14	60.78	13.81
29	60.78	13.76	59.12	13.55	60.96	14	59	13.51
30	61	13.77	60.75	13.83	59.76	13.81	59.02	14.01
31	60.58	13.59	60.66	14	59.12	13.65	60.45	13.62
32	60.76	13.98	60.11	13.78	60.12	13.74	60.35	14.01
33	60.58	13.78	60.3	13.89	59.5	13.52	60.34	13.61
34	59.32	13.52	60.2	13.88	59.78	13.6	60.25	13.61
35	59.31	13.9	60.72	13.99	59.63	13.58	59.3	13.78
36	59.62	14	60.55	13.92	60.12	13.61	59.4	13.63
37	59.55	13.99	60.12	13.95	59.62	13.85	59.63	13.8
38	59.36	13.75	60.15	13.71	59.17	13.71	59.79	13.62
39	60.32	13.66	60.75	13.76	59.36	13.68	59.88	13.57
40	60.38	13.54	60.66	13.8	59.78	13.65	60.35	13.62
41	60.92	13.58	60.55	13.62	59.47	13.77	60.21	13.58
42	60.99	13.77	59.12	13.7	59.62	13.79	60.5	13.65
43	60.58	13.71	59.32	13.52	60.2	13.68	60.76	13.61
44	59.5	13.51	59.3	13.56	60.35	13.81	60.78	13.51
45	59.31	13.63	60.21	13.78	60.78	13.87	60.96	13.51
46	59.45	13.52	60.11	13.66	60.38	13.85	60.32	13.54
47	60	13.66	60.12	13.58	59.78	13.82	59.4	13.58
48	59.5	13.59	59.45	13.54	59.62	13.72	59.33	13.91

49	59.62	13.76	60.12	13.62	59.1	13.8	60.25	13.62
50	59.85	13.55	59.78	13.51	59.3	13.62	60.29	13.91
51	59.72	13.81	60	13.52	60.25	13.71	59.78	13.99
52	59.85	13.62	60.2	13.78	60.34	13.7	60	13.95
53	59.8	13.57	59.45	13.62	60.37	13.59	60.05	13.78
54	60.7	13.82	59.66	13.52	60.1	13.5	60.89	13.62
55	60.85	13.67	60.78	13.61	59.8	13.71	60.04	13.93
56	60.94	13.74	60.12	13.71	59.63	13.68	59.56	13.9
57	60.75	13.69	59.7	13.73	59.45	13.52	59.36	13.66
58	59.63	13.58	59.32	13.51	59.47	13.51	59.48	13.65
59	60.78	13.91	59.6	13.69	60.66	13.68	59.32	13.91
60	60.55	13.67	59.45	13.51	60.01	13.75	60.21	13.89
61	59.81	13.87	59.21	13.82	59.31	13.7	60.13	13.94
62	59.29	13.83	59.32	13.83	59.44	13.57	60.22	13.88
63	61	14	60.91	13.92	60.01	13.69	60.08	13.8
64	60.11	13.92	59.41	14	59.92	13.81	60.17	13.77
65	60	13.77	61	13.63	60.1	13.99	61	13.95
66	59.02	13.58	59.7	13.55	60.24	13.83	60.33	14
67	59.87	13.65	60.05	13.69	60.37	13.67	60.78	13.88
68	60.5	13.7	60.17	13.81	60.59	13.69	59.8	13.67
69	60.71	13.57	60.24	13.97	59.12	13.54	59.76	13.53
70	59.63	13.62	59.12	13.52	59.62	13.92	60.27	13.77
